# Supplementary material for: Transcriptome-wide effects of inverted SINEs on gene expression and their impact on RNA polymerase II activity
Source: Genome Biol. 2016 Oct 25;17:220. doi: 10.1186/s13059-016-1083-0 (PMC5080714; doi:10.1186/s13059-016-1083-0)
Supplement: Additional file 1: — Figure S1. Alu elements in iSINE containing constructs are edited, indicating that iSINEs can form double-stranded structures. Figure S2. Minimum free energy structures of the Znf708 UTR and the designed UTRs. Figure S3. ZNF-analogs mimicking the folding of the wildtype ZNF are edited, indicating that they form double-stranded structures. Figure S4. iSINEs do not lead to nuclear retention of RNAs. Figure S5. iSINE mediated gene repression is independent of dsRNA-activated kinase PKR and DICER or DROSHA activity. Figure S6. iSINEs do not interfere with mRNA polyadenylation. (PDF 2738 kb) [file 13059_2016_1083_MOESM1_ESM.pdf]

Supplementary material

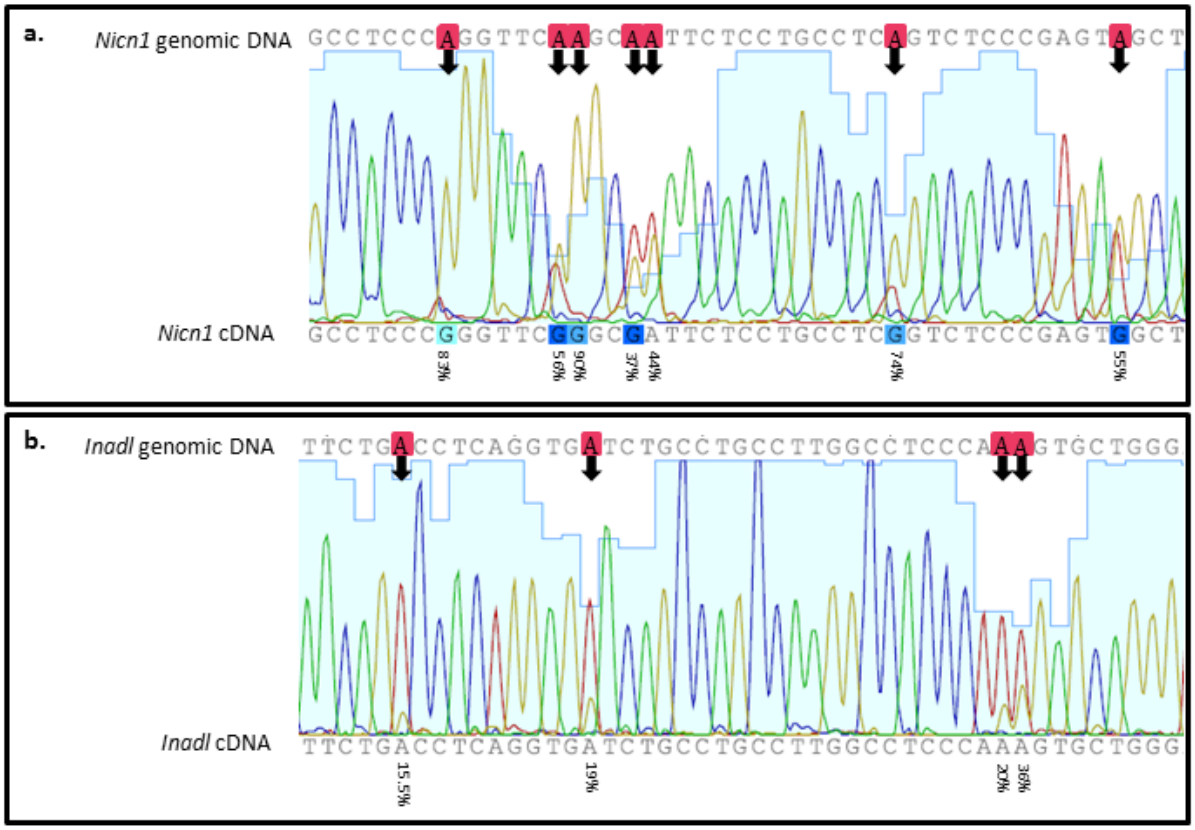

Supplementary Figure S1: Alu elements in *i*SINE containing constructs are edited, indicating that *i*SINEs can form double-stranded structures. a) *Nicn1* and **b)** *Inadl* *i*SINE containing constructs were transfected in wt MEFs, total RNA was extracted, cDNA was synthesized and sequenced. Efficiency of editing was measured by dividing the sum of A and G peak heights by the G peak. The edited adenosine is marked by an arrow and the amount of editing efficiency is shown under the editing peak. Yellow peak resembles G and red peak A residues.

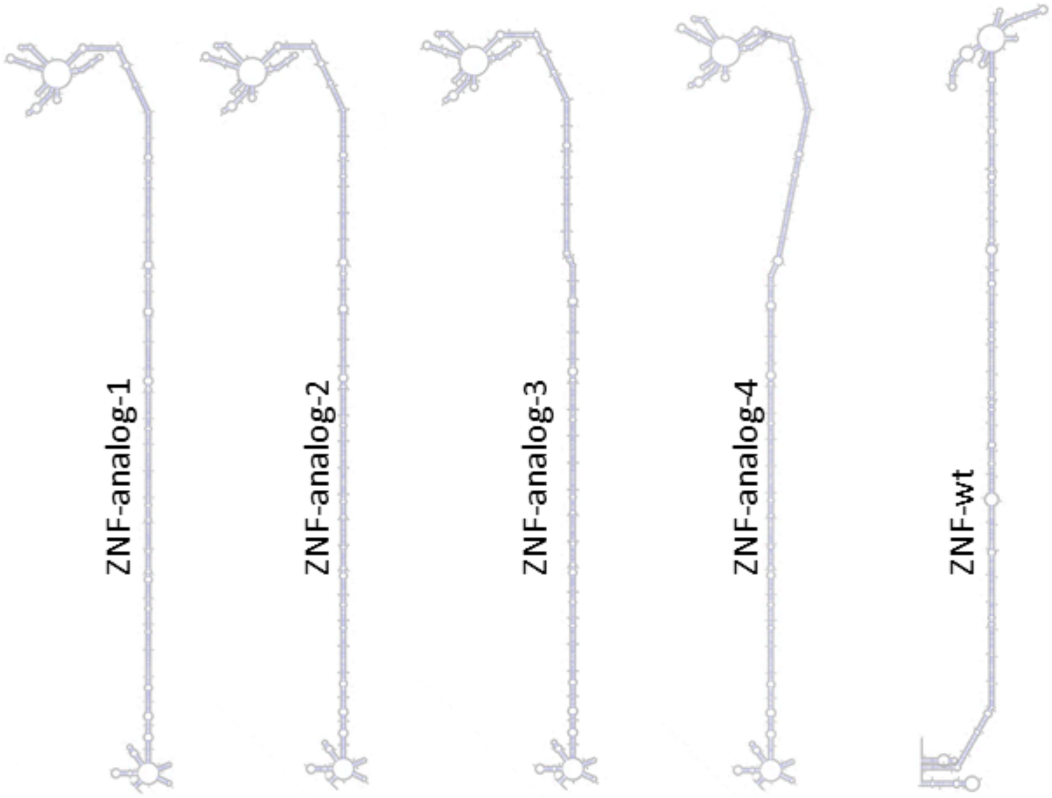

Supplementary Figure S2: Minimum free energy structures of the *Znf*708 UTR and the designed UTRs. The minimum free energy structures of UTRs that resemble the secondary structure of the *Znf*708 UTR are shown. The SINE elements in the UTRs were replaced by non-homologous sequences. As control the original *Znf*708 UTR is depicted as well. For details on the design please refer to the materials and methods section.

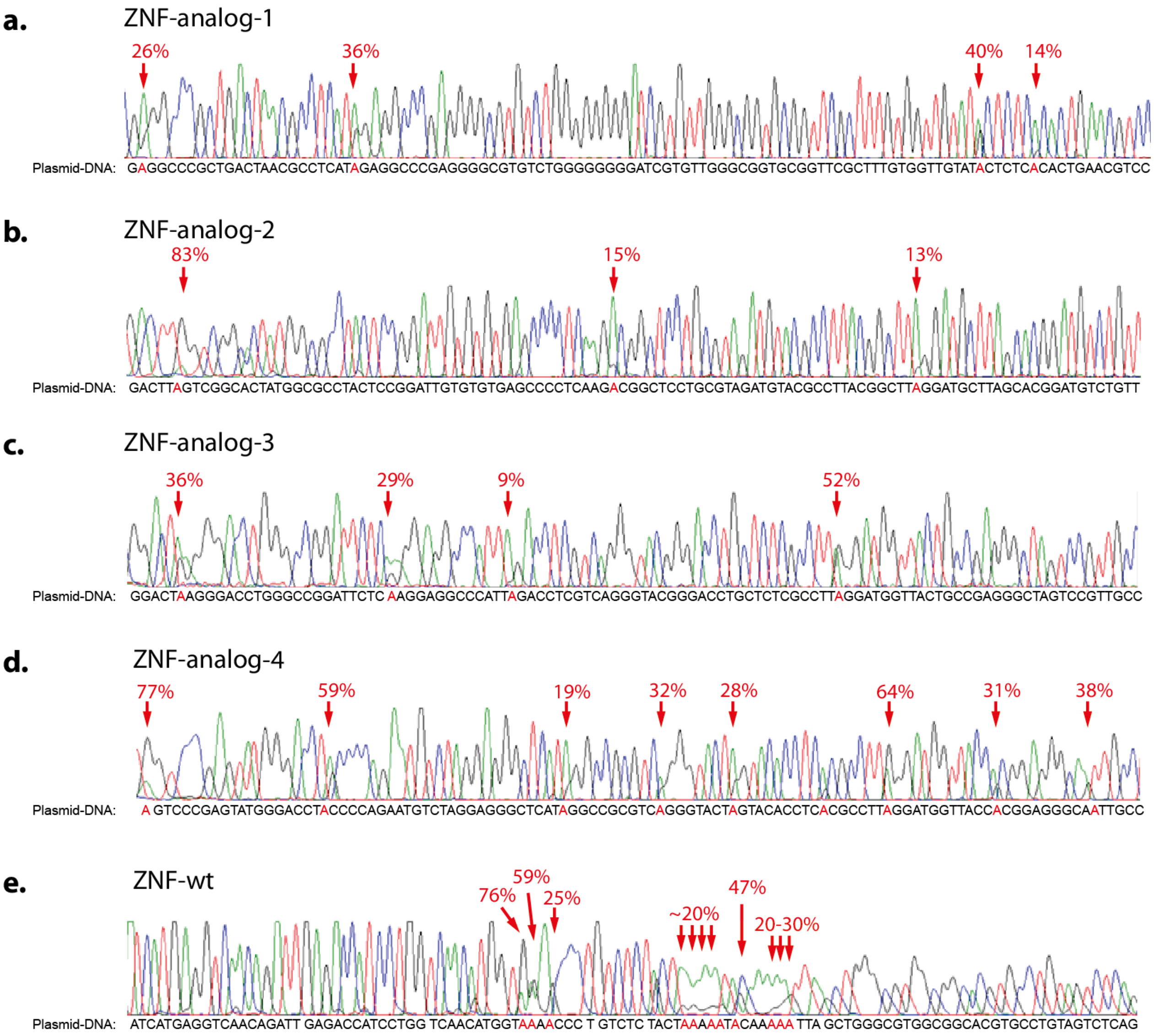

Supplementary Figure S3: ZNF-analogs mimicking the folding of the wildtype ZNF are edited, indicating that they form double-stranded structures.

The ZNF-analogs (see S2) (a-d) and as control the ZNF-wt (e) were transfected into a cell line stably overexpressing ADAR2. Subsequently, RNA was extracted and reverse transcribed and subjected to PCR amplification. Direct sequencing of the respective PCR-products shows that all designed ZNF-analogs as well as the wildtype ZNF reporter are edited to similar extents. Efficiency of editing was measured by dividing the sum of A and G peak heights by the G peak. The edited adenosine is marked by a red arrow and the editing efficiency is given above the peak. All constructs are edited to similar extents and therefore most likely form double-stranded structures of similar extents.

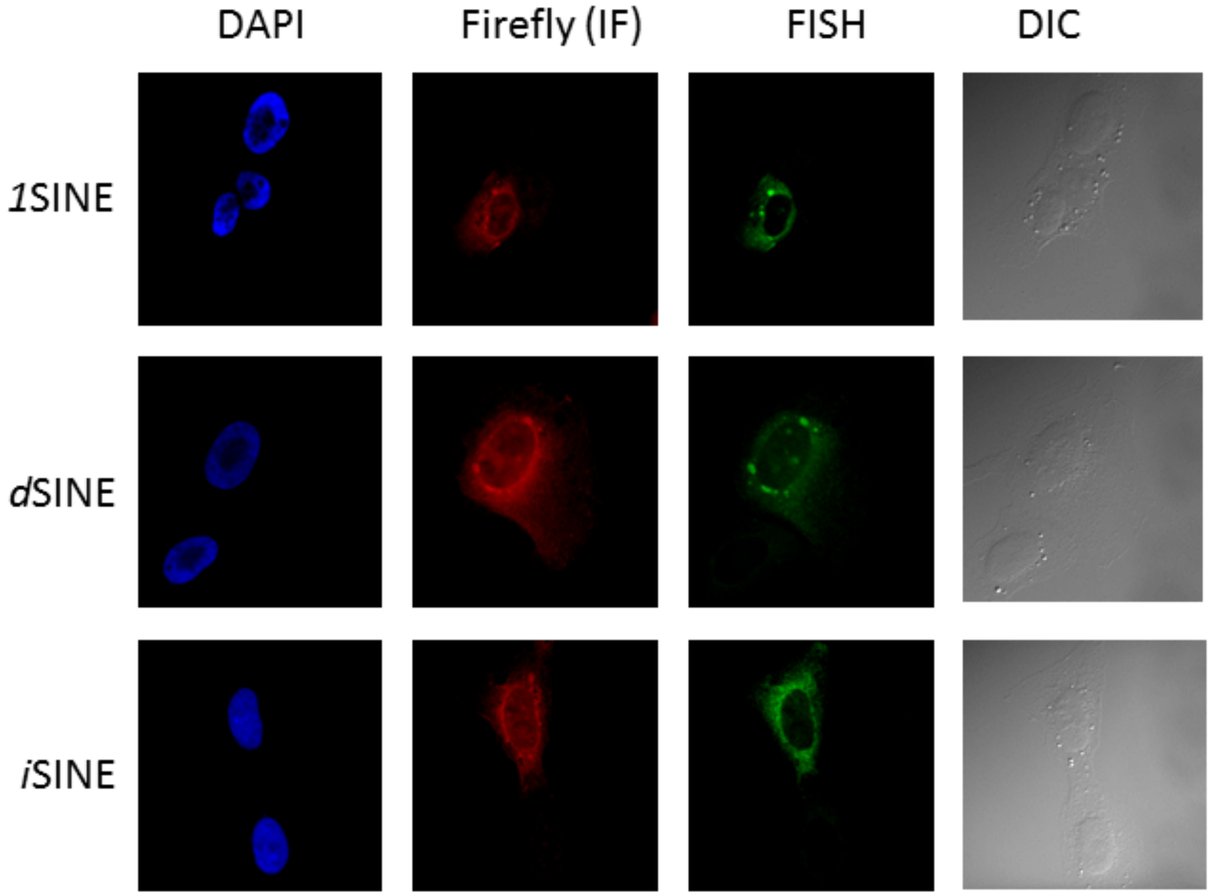

Supplementary Figure S4: *i*SINEs do not lead to nuclear retention of RNAs. U2OS cells transfected with pmiRGLO harboring the *Nicn1 1*SINE, *d*SINE, or *i*SINE downstream of the firefly luciferase ORF were stained for the expression of firefly luciferase (red channel, firefly IF). Localization of the RNA was determined by fluorescent in situ hybridization (FISH) with a probe directed against firefly luciferase (green channel, FISH). Cell nuclei are stained with DAPI (blue channel) while the total cell is visualized by differential interference contrast (DIC).

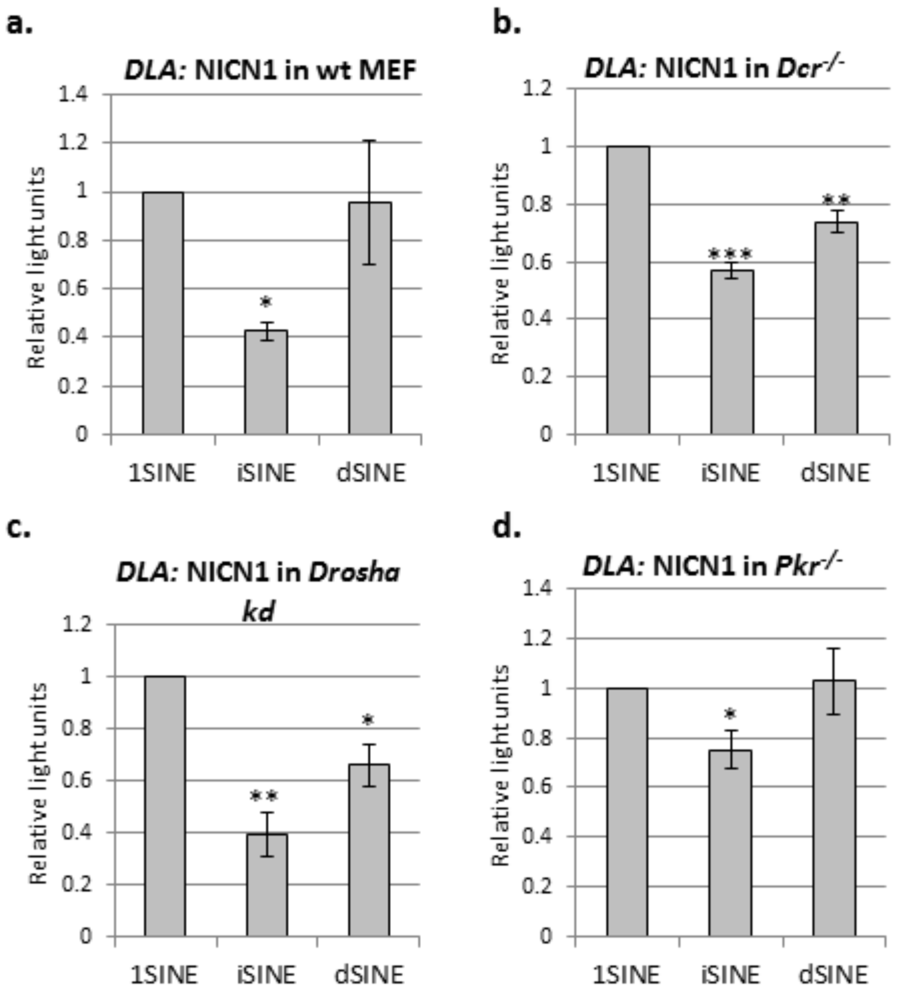

Supplementary Figure S5: *i*SINE mediated gene repression is independent of dsRNA-activated kinase PKR and DICER or DROSHA activity. Reporter constructs were transfected in **a)** wt, **b)** *Dcr*$^{-/-}$ embryonic mouse cells and after 24 hrs gene expression was quantified using a dual luciferase assay. **c)** U2OS cells that constantly express an shRNA against *Drosha* and **d)** *Pkr*$^{-/-}$ embryonic mouse cells were transfected with SINE containing reporter genes. Again, expression was quantified using a dual luciferase reporter assay after 24hrs. Standard deviation is indicated. Asterisks indicate p-values calculated with student's t-test * $p<0.05$, ** $p<0.005$ and *** $p<0.0005$.

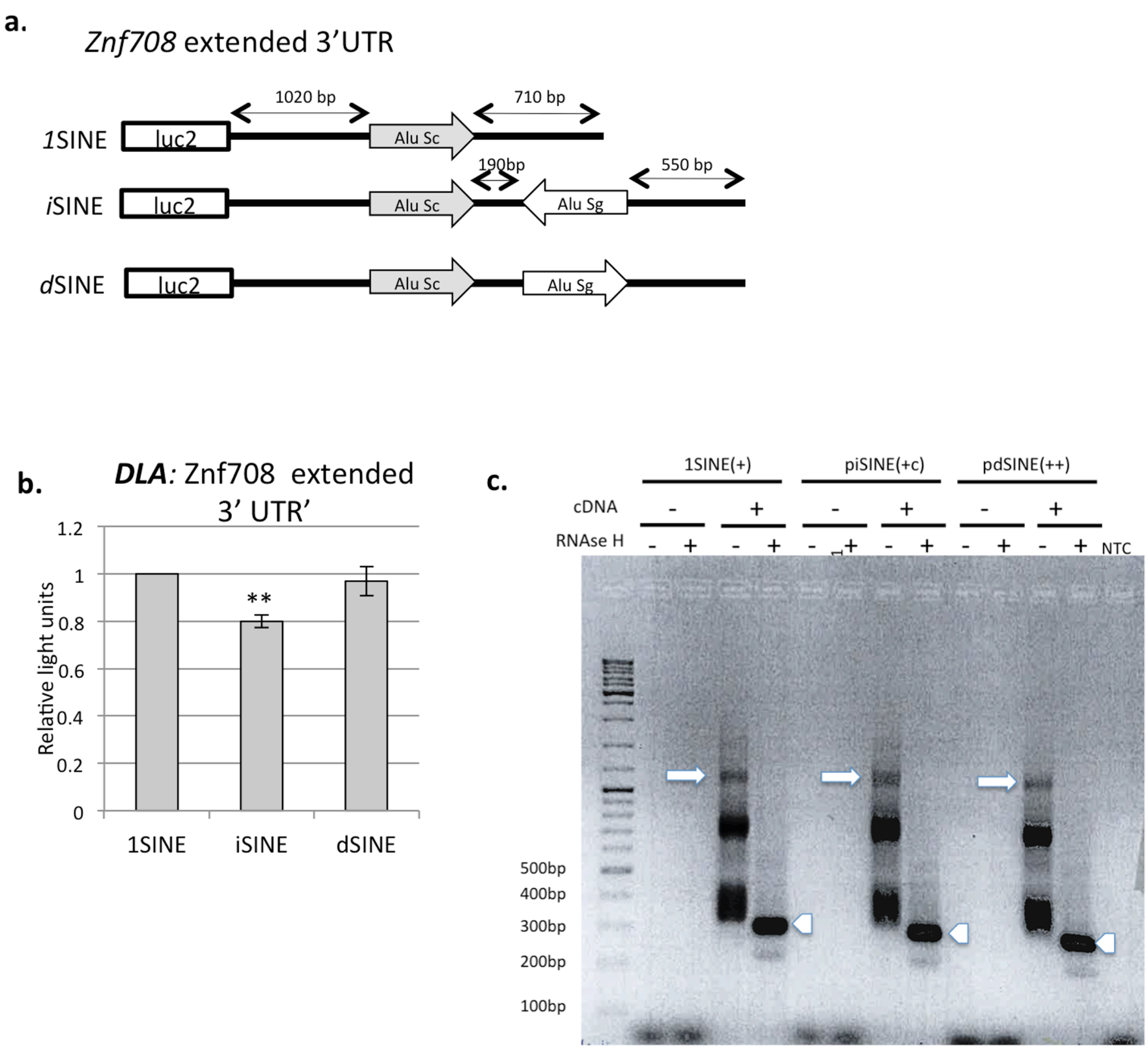

Supplementary Figure S6: *i*SINEs do not interfere with mRNA polyadenylation. a) 950 nucleotides were inserted between the stop codon and the first Alu and 270 nucleotides were introduced between the last Alu and the polyadenylation signal in *Znf708* constructs increasing the distance to 1020 and 550 nucleotides, respectively. **b)** The longer *Znf708* constructs (*Znf708* extended 3' UTR) were transfected in U2OS and a dual luciferase assay was done after 24h. Again, *i*SINEs led to reduced gene expression. p-values calculated with student's t-test ** $p<0.005$. **c)** Poly-

A lengths were determined by polyA tail-splint ligation PCR [1]. Poly A tails (white arrows) and the start of the poly A tail (white arrowheads) are identical in all three constructs.

1. Minasaki R, Rudel D, Eckmann CR: **Increased sensitivity and accuracy of a single-stranded DNA splint-mediated ligation assay (sPAT) reveals poly(A) tail length dynamics of developmentally regulated mRNAs.** *RNA Biol* 2014, **11:**111-123.
