# Supplementary material for: Transcriptome-wide effects of inverted SINEs on gene expression and their impact on RNA polymerase II activity
Source: Genome Biol. 2016 Oct 25;17:220. doi: 10.1186/s13059-016-1083-0 (PMC5080714; doi:10.1186/s13059-016-1083-0)
Supplement: Additional file 2: Table S1. — describing all DNA primers used in this study for regular and quantitative PCR analysis. (PDF 57 kb) [file 13059_2016_1083_MOESM2_ESM.pdf]

Supplementary Table S1: Primer sequences used for qPCR to quantify expression and for the ChIP-experiments.

**qPCR primers for cDNA**		
Renilla	Fw-	AGATCATGCGGAAACTGGAG
	Rev-	CGAAGGTAGGCGTTGTAGTTG
Firefly in pHRLTK	Fw-	GTGCCAACCCTATTCTCCTTC
	Rev-	CCGCTTCCCCGACTTCCTTAG
Firefly in pmirGLO	Fw-	GGCTGAATACAAACCATCGG
	Rev-	CGCTCGTTGTAGATGTCGTTAG
**ChIP primers for Reporter-based assays**		
Nicn1 - Part A	Fw-	TCTGCGGCTTTCGGGTCGTG
	Rev-	CCGCTGGCGATCTCGTGCAAG
Nicn1 - Part B	Fw-	GGCGGCAAGATCGCCGTGTA
	Rev-	GGCCCTATGGGCACACCCAC
Nicn1 - Part C	Fw-	CGGCCGCTTCGAGCAGACAT
	Rev-	TCACTGCATTCTAGTTGTGGTTTGTCC
Znf708 - Part A	Fw-	TCTGCGGCTTTCGGGTCGTG
	Rev-	CCGCTGGCGATCTCGTGCAAG
Znf708 - Part B	Fw-	GGCGGCAAGATCGCCGTGTA
	Rev-	ACCATGCCCGGCCCTACCTC
Znf708 - Part C	Fw-	CGGCCGCTTCGAGCAGACAT
	Rev-	TCACTGCATTCTAGTTGTGGTTTGTCC
**ChIP primers for endogenous targets**		
KIF1B - Part A	Fw-	AATGTGTGGGAGCCTTTTTG
	Rev-	CAGGGGTAGGAATGGAGGAT
KIF1B - Part B	Fw-	GTTGTGGCGGGAAAGTTAAG
	Rev-	TGATGGGAGAAAGGAGGATG
KIF1B - Part C	Fw-	GAGGTTGGTCTCTTGCCAAA
	Rev-	GCTGAGGGACTCCTCAAGTG
UBIAD1 - Part A	Fw-	CATGTTTACCAGCCGGAGTT
	Rev-	GCCACTGGAAGGACAAACAT
UBIAD1 - Part B	Fw-	GGCTGTTTTCCCTTTGTTGA
	Rev-	GGGCTTTGAGTATCCCACAA
UBIAD1 - Part C	Fw-	CTTCCCAGCACCATTTTTGT
	Rev-	CAGAACTGATAGCCCCGATG
OTUD3 - Part A	Fw-	ACACTGTTTTCCCTGGATGC
	Rev-	GGGGAGAGTAAAAATGGCAAA
OTUD3 - Part B	Fw-	CGGGGAGTTGCTCACATATT
	Rev-	CCAGTAAAACCCAGGCAGAG
OTUD3 - Part C	Fw-	CCTCCACCAGCACTAAGCTC

	Rev-	GACGCATGCCAACACTTCTA
SPAG9 - Part A	Fw-	TATTTCCCTTGAGGGGGTTG
	Rev-	AGTACGCAGCACACCATCAG
SPAG9 - Part B	Fw-	CCCCCAAATCTGATGAAAAC
	Rev-	GTCCCGAGAGGGTTAAAAGC
SPAG9 - Part C	Fw-	AACATGGCTGAACAACAGCA
	Rev-	TCTGAGCCATTTTTGGCAAT
AHR - Part A	Fw-	ACCAGGGTTACACTCTTTGTTCA
	Rev-	AAACTTCATGGGCCAAAATG
AHR - Part B	Fw-	AAGCAAAGCACCCATTTCAA
	Rev-	ACTTTGCACCAAACCTTGCT
AHR - Part C	Fw-	TTGTTAAGTGCCAAACAAAGGA
	Rev-	GAAAAACCTGGTACAGGAAAAGA
ATG12 - Part A	Fw-	AAAACGAATCTCCGAACGTC
	Rev-	ACTCCCATTTCCACCTGATG
ATG12 - Part B	Fw-	TTTGAGGTATTCAGCCAGCA
	Rev-	CTGCCTCCAAGGTTGAAAAG
ATG12 - Part C	Fw-	TGTGTGCCATGGGTCATCTA
	Rev-	AAGCCTGGATCCGATAAGTT
DESI1 - Part A	Fw-	TGCAAGTCCCCTCCAGTAAG
	Rev-	GCTGAATTGACCCCAAAGAG
DESI1 - Part B	Fw-	CTTTCTGGGTCACGGATGTT
	Rev-	TGCTGTTTCCTAAGCGTCCT
DESI1 - Part C	Fw-	CTGGCAAATCAGTGGGTCTT
	Rev-	CATCAGCCATCACAGTTTGG
DPY19L4 - Part A	Fw-	TTCAATAGCCGGGCACTATC
	Rev-	GTCCCAAAGGGCTGTTGTAA
DPY19L4 - Part B	Fw-	CCTGTGCTGAATTAGGTTTGG
	Rev-	CCTTTTCAACTGATGCTGGA
DPY19L4 - Part C	Fw-	TGGCATCTGTATCTTGGTGCT
	Rev-	TGCTGCCTATATACACAGAAAGTAAA
